# Factors Associated with Suicidal Ideation and Suicide Attempt in Brazilian Transgender Youth

**DOI:** 10.3390/ijerph20043215

**Published:** 2023-02-12

**Authors:** Ítala Raymundo Chinazzo, Anna Martha Vaitses Fontanari, Angelo Brandelli Costa, Maria Inês Rodrigues Lobato

**Affiliations:** 1 Programa de Pós-Graduação em Psiquiatria e Ciências do Comportamento, Universidade Federal do Rio Grande do Sul (UFRGS), Porto Alegre 90035-003, RS, Brazil; 2 Programa de Pós-Graduação em Psicologia, Pontifícia Universidade Católica do Rio Grande do Sul (PUCRS), Porto Alegre 90619-900, RS, Brazil

**Keywords:** transgender youth, suicide ideation, suicide attempt

## Abstract

The rates of suicidal ideation and suicide attempts among transgender youths are high. However, in Brazil, there are no studies about these outcomes in this population. The present study aims to investigate the prevalence of suicidal ideation and suicide attempts in Brazilian transgender youths (binary and non-binary), in association with predictor variables, following the Minority Stress Theory. The predictor variables analyzed were depressive symptoms, discrimination, gender distress, deprivation, social support, and gender identity support from parents and friends. Participants were recruited through an online survey. The final sample consisted of 213 participants, aged 13 to 25 years old. Two equal regression analyses were performed, one for each outcome. Out of the total, 103 (48.6%) identified as transgender boys, 44 (20.8%) as transgender girls, and 65 (30.7%) as non-binary. The mean age was 18.53 years (SD 2.50). The study found that 57.6% of the sample had depressive symptoms, 72.3% experienced suicidal ideation, and 42.7% had attempted suicide. In the final model, the variables that were associated with suicidal ideation were deprivation, gender distress, and depressive symptoms. As for suicide attempts, the variables deprivation and depressive symptoms were correlated. Further studies on this population should be conducted to analyze protective factors for these outcomes.

## 1. Introduction

The term transgender is used to refer to individuals whose gender identity is not in accordance with their sex assigned at birth, whereas the expression “cisgender” refers to individuals whose gender identity matches their sex assigned at birth [1]. Transgender is an umbrella concept that covers a broad continuum of gender identities, including binary and non-binary people. While binary people identify either as a woman or as a man—transgender or not—non-binary people identify themselves as belonging to a third gender, having more than one gender, having a fluid gender, or being agender [2]. Considering that there are some intersections between non-binary and binary transgender experiences, despite the differences between these identities, we will use the term transgender to designate binary and non-binary transgender identities.

According to the World Health Organization (WHO) [3], the transgender population is considered vulnerable to suicidal behavior. Among the risk factors are depressive symptoms, experiencing conflict and violence, and a sense of isolation [3]. Regarding the younger population, an increase has been perceived in these rates [3], in Brazil as well [4], where 6.41 of every 100,000 people commit suicide, with an increase to 7.61 when considering only youth aged 15–24. In addition, there are high incidences of suicides in low- and middle-income countries such as Brazil. Considering that deaths by suicide can be prevented, studies on this topic are relevant to understand associated factors in the Brazilian transgender youth population.

Studies with young transgender populations have found high rates of depressive symptoms, suicidal ideation, and suicide attempts. A study in New Zealand [5] found a 5.87 times greater risk of transgender youths to attempt suicide compared to their cisgender peers. In the United States, researchers [6] carried out a large epidemiological study with high school students and found high rates of suicidal ideation (43.9%) and suicide attempts (34.6%) in the last 12 months in self-declared transgender adolescents, rates significantly higher compared to their cisgender peers. A meta-analysis [7] including data from the United States, the United Kingdom, Australia, and Japan found a prevalence of 28.0% for suicidal ideation and 14.8% for suicide attempts. In China [8], a survey of young transgender persons found a prevalence of 56.4% for a history of suicidal ideation and 16.1% reported a history of suicide attempts. In Canada [9], a nationally representative population-based study with young people found that transgender adolescents were at an increased risk of suicidality when compared to their cisgender peers, with 5 times the risk of suicidal ideation and 7.6 times the risk of ever having attempted suicide.

In Brazil, there are no studies on transgender youths on suicidal ideation and suicide attempts. A study [10] with Brazilian transgender adults found a prevalence of 67.20% for depressive symptoms, 67.72% for experiencing suicidal ideation, and 43.12% for having attempted suicide. Among those who attempted suicide, 80.50% reported associating their attempt with being a transgender person. Another Brazilian study [11] with transgender adults, based on the medical records of a health clinic, found a prevalence of 73.7% for suicidal ideation, and 29.9% had attempted suicide at some point in their lives. The study found significant differences among young people, with a higher prevalence of 79.2% among those aged 18–20, and 88.7% among those aged 21–25. Another study [12] carried out with binary transgender adults and *travestis* in one Brazilian state found a prevalence of 41.4% for suicidal ideation.

Analyzing the Brazilian social context is important to understand the experiences of the country’s transgender youth. Brazil has high rates of violence against the transgender community, with the highest world rate of homicides of transgender persons according to the Trans Murder Monitoring project [13]. In 2022, 327 trans and gender-diverse people were murdered in the world, with 29% occurring in Brazil, followed by Mexico, with 17.13% [13]. These data are worrying, and evidence part of the violence experienced by the transgender population in Brazil. In this sense, there are Brazilian LGBTQIAP+ organizations that are active in surveying data on these types of violence, as well as social mobilization networks, informative and educational actions to reduce social prejudice and barriers to access transgender rights [14,15].

Public policies have been developed and revised in Brazil, seeking to facilitate transgender people’s access to their rights and to reduce transphobia [16]. The country’s Unified Health System provides gender-affirming procedures free of charge for those seeking body modification, since 2008 in the case of transgender women [17], having been redefined and expanded in 2013 to include other genders [18]. In the case of young people, since 2019 they have been allowed to start hormone therapy from the age of 16, following Resolution No. 2265 of the National Council of Medicine [19]. Legally, since 2018, it is possible to rectify name and gender in national documents without undergoing legal procedures for those over 18 years of age [20]. However, barriers to accessing these services and rights continue to exist in practice, marked by stigma and prejudice [21,22]. Among the difficulties, for example, there are few health services providing comprehensive health care for the trans population, concentrated mainly in the south and southeast regions, despite the country’s large geographic extension.

As stated earlier, there are mental health disparities when compared with the young cisgender population [5,6,9]. Some disparities can be associated with stigma, harassment, and victimizing experiences of transphobia. The Minority Stress Theory (MST) understands these mental health disparities as the stress experienced by people belonging to minority groups, who experience, in addition to the usual stress of daily life, enacted stigma, expectations of rejection, and internalization of social stigma [23,24,25,26]. A stigmatizing social environment leads transgender populations to experience gender and socially based stress, which can be distal (objective stressors, discrimination, victimization) or proximal (subjective stressors, internalized transphobia, expectations for future prejudice events). Specifically, regarding suicide, there are other theories, such as the Interpersonal Theory of Suicide (IPTS), developed by Joiner and Van Orden [27], which have also been used to understand this outcome in the transgender community [28,29,30]. There seems to be compatibility between the theories due to their focus on interpersonal factors, such as social rejection [28]. IPTS understands lethal and nonlethal behaviors associated with suicide as arising from severe interpersonal emotional conflicts, based on thwarted belongingness and perceived burdensomeness [28,29,30].

On the other hand, there are protective factors for these mental health disparities, such as minority resilience factors. According to the MST [26], identifying as part of a minority group may give access to community resources such as support groups, community centers, minority role models, as well as affirmative actions and specialized clinics. Among the protective factors for mental health, both the MST and ITPS indicate social support and gender identity support as mechanisms to buffer the harmful effects of prejudice. For the IPTS [28], supportive interpersonal relationships for transgender youth play an especially important role, in view of gender explorations that are specific to this age group. Transgender youth can explore their gender identity and seek support when they feel they belong [29,30]. However, young transgender persons report conflicts with family and peers due to their transgender identities [29,30].

The present study aims to investigate the prevalence of suicidal ideation and suicide attempts in young Brazilian transgender people (binary and non-binary) and predictors of risk and protection for the outcomes according to some concepts from the MST and IPTS. We do not aim to measure all the concepts of both theories (MST, IPTS) or compare the theories with each other; we have used both theories as a theoretical basis to interpret the results. There are no Brazilian studies with this population, so our objective is to get to know this population and understand whether there are differences between binary and non-binary genders in terms of sociodemographic characteristics and outcomes, and which risk and protective factors may be associated with the outcomes. As risk factors, depressive symptoms, discrimination, gender distress, and deprivation were evaluated. For protective factors, gender positivity, general social support, and gender identity support from parents and friends were analyzed.

## 2. Materials and Methods

### 2.1. Study Sample

Data were collected online in 2018 through Facebook invitations sent to young Brazilians who “liked” pages or joined groups linked to the LGBTQIAP+ movement. To measure transgender gender identity, the Two Steps Method [31,32] was used, which consists of two questions. First, we asked about the sex assigned at birth recorded on the birth certificate, with “female” and “male” as possible answers. The second question was about the gender identity with which the participant currently identified. We categorized the second answer into “woman,” “man,” and “non-binary.”

**Figure 1:**
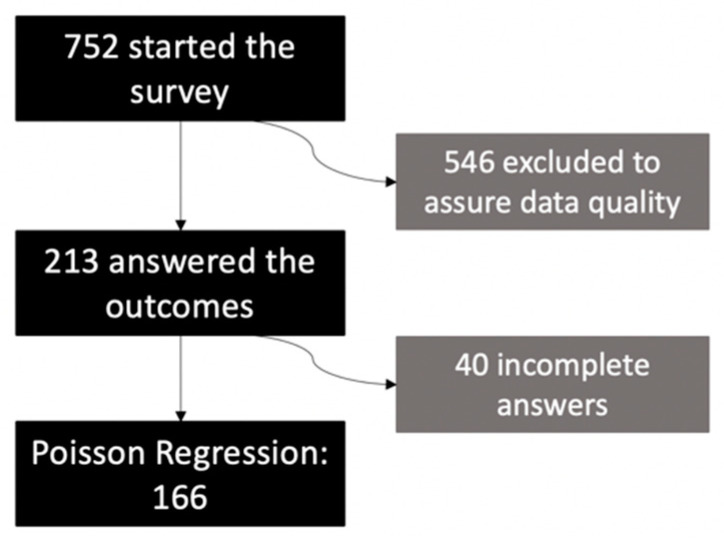
Participants sampling flowchart.

As the study was carried out from an online survey, some actions were taken to ensure the quality of the sample data. Likewise, duplicates and discrepant responses were removed, and those who did not answer both questions of the two-step questions as transgenders were not included in the sample. In total, 752 people started the survey, however, only 213 people responded to the outcomes and variables of our study. Of these, 166 were included in the statistical analyses due to complete responses to the scales used in the study. Figure 1 summarizes the participants’ flowchart.

### 2.2. Measures

The survey was inspired by the TransYouth CAN! Project (see https://transyouthcan.ca/). The cross-cultural adaptation for the Brazilian population was based on the work by Borsa, Damásio, and Bandeira [33]; it is better described in previous publications [34].

The outcomes of “suicidal ideation” and “suicide attempt” were measured with “yes” or “no” questions. The first question was “Have you seriously considered committing suicide or taking your own life?”, and whether this had happened in the last 12 months. The second outcome was addressed with the question “Have you ever tried to commit suicide or tried to take your own life?”, and whether this had happened in the last 12 months.

Socioeconomic Status (SES) was measured using the Deprivation Scale, created by the TransYouth CAN! Team to access information about whether our sample had their basic needs met. The Deprivation Scale consisted of five questions on whether participants had access to school supplies, internet, season-appropriate clothing, other essential clothing, and trustworthy transportation in a one-year period. Each question could be answered using a five-point Likert scale ranging from “never” (1) to “always” (5), in which a higher score represents less deprivation. The score was calculated by the arithmetical mean of the five questions. The Cronbach’s alpha was 0.84.

The Modified Depression Scale (MDS) [35] was used to assess the frequency of five depressive symptoms in a 30-day period: felt very sad, felt very angry or in a bad mood, felt hopeless about the future, slept much more or much less than usual, and had difficulty concentrating on studies. The scale was validated in 2012 by Dunn, Johnson, and Green [35]. Each question was answered on a five-point Likert scale, from “never” (1) to “always” (5). The responses were dichotomized according to the cut-off point of 17, in which participants who scored more than 17 were more likely to receive a diagnosis of depression. The Cronbach’s alpha was 0.80.

Discrimination was assessed by the Lifetime and Daily Discrimination Subscale (InDI-D) of the Intersectional Discrimination Index, created and validated by Scheim and Bauer [36]. The answer alternatives were dichotomous, yes or no. The subscale consists of 9 items, addressing forms of prejudice experienced by trans people for being who they are, asking whether they had heard jokes about them; been treated as if they were hostile, useless, or rude; been called unpleasant names; been treated as if they were threatening; been looked at or pointed out in public; been told to think, act, or look more like others; heard that people like them are not normal; been asked offensive, inappropriate, or very private questions; been treated as if they were less intelligent. For the analyses, the score used was mean, indicating the variability of forms of prejudice that the person experienced. The Cronbach’s alpha was 0.75.

The Multi-Dimensional Scale of Perceived Social Support was used to measure social support. The scale is adapted for the Brazilian population [37], composed of 19 questions divided into four dimensions—affective support, positive social interaction support, emotional/information support, and material support. The question is about how often each type of support is available to the participant when they need it. Some examples of questions from each dimension are “someone who shows you love and affection” (affective), “someone to do something nice with you” (positive social interaction), “someone to trust or talk about yourself or your problems” (emotional/information), and “someone to help with daily tasks if you were sick” (material). It does not ask about the people they can count on, but the frequency of types of support when needed. Responses are given on a Likert scale from 1 (never) to 5 (always). For the analyses, the score used was the mean of each dimension of the scale. The Cronbach’s alpha was calculated by dimension: affective support was 0.92, positive social interaction support was 0.97, emotional/information support was 0.96, and material support was 0.84.

Regarding social support in relation to gender identity, we asked how much certain people support the participant’s gender identity and expression. Responses were measured on a Likert scale from 1 (no support) to 4 (very supportive)—5 (does not apply to me). Support was separated according to relationships—father support was considered the support of a paternal figure (biological father, adoptive father, or stepfather); mother support was considered the support of a maternal figure (biological mother, adoptive mother, or stepmother); and friends support included a sum of friends (cisgender, transgender, lesbian, gay, bisexual, online friends, and classmates). In the final model (Poisson regression), support from fathers and mothers was grouped into a single variable called Parents’ Support of Gender Identity; the variable referring to the support received by friends was called Friends’ Support of Gender Identity.

To evaluate gender distress, the TYC-GDS (Gender Distress Scale) was used, a scale created by the Trans Youth Can! Team, appropriate for binary and non-binary persons [38,39]. The scale consists of 15 items, covering distress related to the body (e.g., I wish I had been born in a different body) and social life (e.g., It bothers me to be addressed as the wrong gender). The items were answered on a five-point Likert scale, ranging from “totally disagree” (1) to “totally agree” (5), plus the answer option “does not apply to me.” The score was obtained by summing up all items; for analysis, the answers were divided into whether the participants identified as experiencing no or almost no gender distress, experiencing gender distress, or experiencing a lot of gender distress. The Cronbach’s alpha was 0.82.

Gender Positivity was also measured using a scale developed by the Trans Youth Can! Team, the TYC-GPS (Gender Positivity Scale) [38,39]. The scale is composed of 12 items comprising matters related to gender pride (I see being trans as a quality; I take pride in expressing my true gender), body pride (I feel confident in my body; I feel attractive), and pride associated with social life (I feel happy when society recognizes me externally the same way I recognize myself internally; I feel approval when strangers in public address me as my true gender). They answered on a Likert scale, from 1 (strongly disagree) to 5 (strongly agree), plus the option “does not apply to me.” The Cronbach’s alpha was 0.78.

### 2.3. Statistical Analysis

The software SPSS 18.0 was used to perform the statistical analyses. In order to describe the sample, in addition to the descriptive analyses, comparisons were also made between the variables according to gender (trans men, trans women, and non-binary) using the chi-square and ANOVA tests. First, background characteristics were individually correlated using Poisson regression with both suicidal ideation and suicide attempt outcomes in the last 12 months. Subsequently, those variables that were statistically significant were included in the final model for multivariate Poisson regression. The excluded variables, to both outcomes, were friends’ support for gender identity, affective support, positive social interaction support, and emotional/information support. Two equal regression analyses were performed, one for each outcome. In the first stage, they considered deprivation and material support. In the second step, the variables discrimination, gender distress, gender positivity, and parents’ support of gender identity were included. Finally, in the third step, depressive symptoms were included in Poisson regression analyses.

### 2.4. Ethical Considerations

The Ethics and Research Committee of the Federal University of Rio Grande do Sul Psychology Institute approved this project (14221513.4.0000.5334). All participants signed an online informed consent form; for participants aged under 18 years, signed consent was provided by their guardians. Participants signed an informed consent form to participate after being told about the objectives of the study and possible harm from the questionnaire, such as embarrassment and sadness when recalling traumatic events. Participants were informed about voluntary participation and being able to withdraw at any time.

## 3. Results

The sample is composed of 103 trans men (48.6%), 44 trans women (20.8%), and 65 non-binary trans people (30.7%), who lived mainly in the south (29.9%) and southeast (47.3%) of Brazil, mainly in the capitals (80.7%) (Table 1). There were no significant differences between the variables according to gender.

The sample showed high rates of mental health variables, with no significant differences depending on gender. Among the participants, 72.4% had experienced suicidal ideation, 42.4% had attempted suicide, and 57.6% had depressive symptoms (Table 2). Table 3 describes the means of the predictor variables. The mean age of the sample is 18.52 years (SD 2.50), ranging from 13 to 25 years old. Among these variables, there were significant differences found for gender distress and age. Regarding gender distress, post hoc comparisons using the Tukey HSD test indicated that the mean score for non-binary persons (M = 3.57, SD = 0.67) was significantly lower than for trans men (M = 3.98, SD = 0.46) and trans women (M = 4.03, SD = 0.53). However, trans men did not significantly differ from trans women. Regarding age, the post hoc Tukey HSD test indicated that trans women (M = 19.91, SD = 2.81) were significantly older than the trans men (M = 18.16, SD = 2.42) and non-binary persons (M = 18.17, SD = 2.11). However, trans men did not significantly differ from the non-binary participants.

All predictor variables were analyzed for both outcomes, suicidal ideation, and suicide attempt, individually. Only those that were statistically significant were included in the final model of each Poisson regression analysis. The final model of both outcomes included the same variables—deprivation, discrimination, gender distress, gender positivity, parents’ support for gender identity, and material support. The variables friends’ support to gender identity, affective support, positive social interaction support, and emotional/information support were excluded.

Table 4 presents the three steps of the Poisson regression for the suicidal ideation outcome. In the third stage, the variables statistically associated with suicidal ideation were deprivation, gender distress, and depressive symptoms. It is important to highlight that more variables (discrimination and gender positivity) were associated with the outcome in the second step; however, they lost statistical significance with the inclusion of depressive symptoms in the model.

As for the suicide attempt (Table 5), in the third step, the variables deprivation and depressive symptoms were significantly associated with the outcome. The discrimination variable loses associative strength from the second step to the third, maintaining a borderline association. In the second step, gender distress presents a borderline association.

## 4. Discussion

The sample showed worrying mental health rates. Of the participants, 72.3% had seriously thought about taking their own life, 42.7% had attempted suicide, and 57.6% had depressive symptoms. The high rates found in this Brazilian sample are in line with those found in other countries, such as the United States [6,7,40,41], China [8], Canada [9], the United Kingdom [7], Australia [7], Japan [7], and New Zealand [5]. However, in China [8], in a binary sample, trans women had significantly higher rates of mental illness than trans men, and our study found no significant difference between genders. We emphasize that this is not a clinical sample, and we call attention to the mental health care of young transgender persons in Brazil. Health professionals, especially mental health professionals, must be aware of the psychic suffering in this population, which, in addition to everyday stressors, also suffers from gender-based minority stressors. The sample showed no difference between binary and non-binary transgender people. We suggest that future Brazilian research continue to investigate differences between gender identities. There are reports of the invisibility of non-binary identities, which could worsen mental health outcomes in this population [42,43,44,45]. In Brazilian reality, the Portuguese language is formally binary, so the neutral pronoun is seldom used. In Brazil, even with resistance, some institutions are adhering to the neutral pronoun as a strategy for the inclusion and belonging of the non-binary transgender population.

Both study outcomes, suicidal ideation, and suicide attempt showed some similar associations with the predictor variables. In the third step of the Poisson regression, the variables deprivation and depressive symptoms were significantly associated with both outcomes. Most of the sample (57.6%) reported experiencing depressive symptoms, which were significantly associated with suicidal ideation and suicide attempts. There were no significant differences between genders. The association between depression and suicide is already known in the general population [3], and in the transgender population [40]. A study [40] in the United States found an association between depressive symptoms and victimization associated with suicidal ideation in trans and gender-diverse youth. The transgender youth population presents aggravating factors such as minority stressors, experiencing gender-based prejudice, and victimization. In our Brazilian sample, the presence of depressive symptoms interfered with the relationship between discrimination and suicidal ideation and suicide attempt, which we suggest should be further researched. However, our results indicate the relevance of clinic and care interventions targeting depressive symptoms to reduce the outcomes.

Regarding experiences of discrimination, the sample presented a high mean of 7.59 (SD 1.82), considering the maximum score of the scale to be 9. This result indicates that the sample had suffered from various forms of day-to-day prejudice (nicknames, jokes, pejorative judgments) throughout their lives; however, it does not indicate how often and when the person suffered this violence. These experiences of prejudice stemming from the fact that they are trans people, suggesting situations of explicit transphobia. In a study with Brazilian transgender adults [10], there was no association found between perceived prejudice (objective) and suicidal ideation and suicide attempt, but there was between proximal stressors and outcomes. Perhaps the association between prejudice and suicidal ideation is mediated by proximal stressors [28]; according to the MST [23,24,25,26], proximal stressors result from experiences of prejudice, and distal stressors result from discrimination. Even though in this sample there was no direct relationship found between day-to-day discrimination, suicide ideation, and suicide attempt, we emphasize the importance of actions aimed at reducing the prejudice experienced by young transgender people.

In our sample, regarding gender distress, there was a significant difference between binary and non-binary transgender identities, and it was only associated with suicidal ideation in the final analysis. In the present study, this variable does not represent the diagnosis of Gender Dysphoria as provided by DSM 5 [46]. It is gender distress as a symptom of psychological distress [47], measured continuously to determine intensity [48], including matters linked to their social life (e.g., being hurt by being called the wrong name/pronouns) and their body (e.g., wishing they were born in a different body). In our sample, those with binary identities (men and women) showed greater gender distress than non-binary participants, which we suggest should be better investigated by differentiating the dimensions of gender distress, social life, and body. Actions to reduce gender distress are often associated with gender-affirming processes, including, for example, ID documents according to the person’s chosen name and the use of gender-affirming hormonal therapy. The process of gender affirmation is subjective and recommended for the reduction of social and bodily stresses, and should focus on the patient’s personal goals, according to their understanding of gender [2,49]. Another study by our research group [50] with a sample of transgender youths found that most gender-affirming processes were significantly associated with experiencing less anxiety and depressive symptoms. There is little literature specifically on non-binary transgender experiences in relation to gender distress and gender-affirming processes, which are historically studied from the binary transgender population point of view [2], but guidelines have been updated to include specific attention to non-binary identities [49,51].

Also noteworthy is the association of gender positivity and suicide ideation in the second step of Poisson regression, and the loss of associative strength with the addition of the variable depressive symptoms to the analysis. This variable counteracts gender distress and includes positive feelings about gender. It included personal views (“I feel proud of being able to express my true gender”) and social ones (“I feel approval when strangers in public address me with my true gender”). The sample had a mean of 3.34 (SD 0.59) for gender positivity, on a scale with a maximum score of 5; we suggest that affirmative actions and positive feelings be encouraged in young people, who may feel more encouraged to explore their gender identities. As discussed by the MST, perhaps gender positivity can be encouraged through community connectedness, such as minority resilience [26,28,45]. According to the IPTS [28,29,30], a feeling of belonging can be cited as positive relations, through inclusive relations and not rejections. It may be considered an important variable for mental health, and we suggest that it be further investigated in Brazil, along with other predictor variables of mental health. In another study [50], gender positivity was associated with better mental health outcomes, less anxiety, and depressive symptoms.

Another common characteristic among the outcomes was the lack of association with supporting variables. The general social support questionnaire is used for the general population, addressing support in different life contexts (e.g., having someone to talk to, someone whose advice I want to hear, someone to help me when I’m bedbound). According to the MST [23], there are particularities in transgender experiences, for which other supports are more relevant to reduce suicidal ideation and suicide attempts. However, the specific variables of support for trans identities, by parents and friends, also did not associate with the outcomes assessed in this sample. This result is surprising in view of the MST and IPTS, predicting social support as a protective factor for mental health. Furthermore, another Brazilian study found an association between social support variables and suicide ideation and suicide attempt for transgender adults [10]. For this Brazilian sample, perhaps the negative variables, such as deprivation and discrimination, had more impact on the outcomes, so the positive ones, such as gender support, social support, and gender positivity, did not reach associative strength to buffer the outcomes. The focus, in the Brazilian context, is still on minimizing the negative effects of experiences of discrimination and gender distress, beyond strengthening positive aspects such as gender positivity. Still, we recommend social support and gender support as mental health tools for the young transgender population.

Our study reiterates the logic of comprehensive, affirmative and respectful health care for transgender youths (binary and non-binary), including professionals from health services, schools, and other relevant settings [52]. Actions must promote safe and reliable spaces for gender exploration, through an inclusive and positive outlook. Gender diversity-friendly environments reduce minority stressors and adverse effects on mental health [49]. In addition, relationships without rejection, according to the IPTS [28], can reduce suicidal ideation and suicide attempts. In the literature [53,54,55], rejection by family, friends, and colleagues is associated with negative outcomes, including suicidal ideation and suicide attempts. Our study investigated support for the transgender identity of young people by friends and parents. We suggest that these relationships be further explored in future research, seeking to understand which specific types of support for transgender identity and gender experiences can reduce suicidal ideation and suicide attempts among young people.

## 5. Limitations

The study presented results from an online sample, which may indicate a bias in the selection of participants, for example, the study sample is mostly characterized by youths residing in urban areas. However, it managed to include participants from different regions of Brazil, a country with a wide geographic territory, despite not being a significant sample of the entire Brazilian transgender population. It also expanded the sample to those who are not linked to health services, covering young trans persons who do not want and/or who have not yet reached health services. Considering the barriers to accessing health services, it is assumed that trans youths who receive health care may have more social support. However, we suggest future research comparing clinical and non-clinical samples, also investigating the relationship between affirmative gender care and the mental health of transgender youths.

Important variables for the MST and IPTS were not analyzed in the study. For a better understanding of the theories with the young Brazilian trans population, we suggest further studies including these variables. In addition, considering the lack of significant association of social support variables with both outcomes (suicidal ideation and suicide attempt), we suggest further studies to understand the perception of general social support and gender social support by young Brazilian transgender people. We suggest that the variables of general social support and support for trans identity continue to be investigated by Brazilian researchers to better understand how to decrease the suffering of Brazilian trans youths, especially in relation to suicidal ideation and suicide attempts. Considering the high prevalence of both outcomes in this sample, it is important to better explore protective factors to create more effective interventions in mental health care for the trans youth population. Additionally, future research could investigate differences between age cohorts, as well as between genders (men, women, non-binary) and potential intersectionality effects (class, ethnicity, disability).

## 6. Conclusions

In the sample, we found similar predictor variables for suicidal ideation and suicide attempts. The same model was analyzed for both outcomes, and both are significantly worsened in the presence of depressive symptoms and with greater deprivation. Suicidal ideation is also intensified with a greater presence of gender distress. The findings help to improve the health services offered to this population, especially considering the high rates of depressive symptoms, suicidal ideation, and suicide attempts in this population. We alert professionals from different settings in the lives of young people (schools, health services, social organizations, family relationships) to this reality, directing comprehensive care to transgender young people, protecting them from discrimination and offering support. We recommend affirmative approaches as support strategies and interventions in health, validating the gender identities of young people and establishing interpersonal bonds of acceptance.

## Figures and Tables

**Table 1:** Sample description by gender.

	Trans Men	Trans Women	Non-Binary	χ^2^	df	*p*	Total
**Live in**							
Capital	77 (77.8%)	39 (88.6%)	51 (79.7%)	3.193	4	0.526	167 (80.7%)
Metropolitan region	18 (18.2%)	4 (9.1%)	12 (18.8%)				34 (16.4%)
Countryside	4 (4.0%)	1 (2.3%)	1 (1.6%)				6 (2.9%)
**Brazil Region**							
Southeast	45 (46.4%)	19 (44.2%)	31 (50.8%)	2.939	8	0.938	95 (47.3%)
South	32 (33.0%)	11 (25.6%)	17 (27.9%)				60 (29.9%)
Other	20 (20.7%)	13 (30.3%)	13 (21.4%)				46 (22.9%)
**School**							
Public school	50 (48.5%)	22 (50.0%)	28 (43.1%)	2.549	6	0.863	100 (47.2%)
Private school	17 (16.5%)	8 (18.2%)	8 (12.3%)				33 (15.6%)
Homeschooled	2 (1.9%)	1 (2.3%)	2 (3.1%)				5 (2.4%)
Not studying	34 (33.0%)	13 (29.5%)	27 (41.5%)				74 (34.9%)

**Table 2:** Mental health variables by gender.

	Trans Men	Trans Women	Non-Binary	χ^2^	df	*p*	Total
**Suicidal Ideation**							
No	26 (26.0%)	16 (38.1%)	14 (23.0%)	3.104	2	0.212	56 (27.6%)
Yes	74 (74.0%)	26 (61.9%)	57 (77.0%)				147 (72.4%)
**Suicidal Attempt**							
No	56 (56.6%)	26 (60.5%)	35 (57.4%)	0.189	2	0.910	117 (57.6%)
Yes	43 (43.4%)	17 (39.5%)	26 (42.6%)				86 (42.4%)
**Depressive Symptoms**							
No	37 (36.6%)	23 (53.5%)	29 (45.3%)	3.741	2	0.154	89 (42.8%)
Yes	64 (63.4%)	20 (46.5%)	35 (54.7%)				119 (57.2%)

**Table 3:** Predictor variables of the final model by gender.

	Trans Men	Trans Women	Non-Binary	F	*p*	Total
**Deprivation**	4.37 (0.69)	4.10 (0.85)	4.35 (0.69)	2.256	0.107	4.31 (0.73)
**Age**	18.16 (2.42)	19.91 (2.81)	18.17 (2.11)	9.098	**0.000** *	18.52 (2.51)
**Discrimination**	7.45 (1.87)	7.79 (1.68)	7.63 (1.86)	0.521	0.595	7.58 (1.82)
**Gender Distress**	3.98 (0.46)	4.03 (0.53)	3.57 (0.67)	12.179	**0.000** *	3.88 (0.57)
**Gender Positivity**	3.26 (0.54)	3.40 (0.64)	3.43 (0.65)	1.709	0.184	3.34 (0.60)
**Parents’ Support to GI**	2.43 (1.13)	2.75 (1.31)	2.40 (1.15)	1.395	0.250	2.49 (1.18)
**Friends’ Support to GI**	3.47 (0.53)	3.41 (0.57)	3.31 (0.61)	1.498	0.226	3.41 (0.57)
**Social Support**						
Affective	11.31 (3.56)	10.79 (4.58)	10.52 (3.98)	0.817	0.443	10.96 (3.92)
Positive Social Interaction	13.98 (5.00)	13.38 (5.49)	12.63 (5.55)	1.235	0.293	13.43 (5.28)
Emotional/Information	26.56 (9.83)	26.78 (10.26)	26.22 (9.81)	0.045	0.956	26.50 (9.87)
Material	13.29 (4.51)	12.90 (5.00)	12.86 (4.69)	0.196	0.822	13.07 (4.65)

**Bold:** * *p* < 0.00; GI, gender identity.

**Table 4:** Poisson Regression—suicidal ideation.

	Step 1				Step 2				Step 3			
	*p*	Exp (B)	CI (95%)		*p*	Exp (B)	CI (95%)		*p*	Exp (B)	CI (95%)	
**Deprivation**	**0.00** *	0.85	0.77	0.95	**0.04** *	0.90	0.81	1.00	**0.05** *	0.90	0.82	1.00
**Material Support**	0.15	0.99	0.97	1.01	0.77	1.00	0.98	1.02	0.25	1.01	0.99	1.03
**Discrimination**	-	-	-	-	**0.02** *	1.09	1.02	1.17	0.09	1.06	0.99	1.14
**Gender Distress**	-	-	-	-	**0.01** *	1.29	1.07	1.56	**0.01** *	1.27	1.06	1.52
**Gender Positivity**	-	-	-	-	**0.03** *	0.82	0.68	0.98	0.23	0.89	0.75	1.07
**Parents’ Support to Gender Identity**	-	-	-	-	0.39	0.96	0.88	1.05	0.37	0.96	0.88	1.05
**Depressive Symptoms**	-	-	-	-	-	-	-	-	**0.00** *	1.04	1.02	1.06

**Bold:** * *p* < 0.05; CI, confidence interval.

**Table 5:** Poisson Regression—suicide attempt.

	Step 1				Step 2				Step 3			
	*p*	Exp (B)	CI (95%)		*p*	Exp (B)	CI (95%)		*p*	Exp (B)	CI (95%)	
**Deprivation**	**0.01** *	0.77	0.63	0.95	0.09	0.84	0.68	1.03	**0.05** *	0.81	0.67	1.00
**Material Support**	0.31	0.98	0.94	1.00	0.73	1.01	0.97	1.05	0.31	1.02	0.98	1.06
**Discrimination**	-	-	-	-	**0.05** *	1.17	0.99	1.37	0.07	1.14	0.99	1.31
**Gender Distress**	-	-	-	-	0.06	1.39	0.98	1.99	0.16	1.24	0.91	1.81
**Gender Positivity**	-	-	-	-	0.09	0.75	0.53	1.05	0.23	0.82	0.60	1.13
**Parents’ Support to Gender Identity**	-	-	-	-	0.49	0.95	0.81	1.11	0.67	0.97	0.83	1.13
**Depressive Symptoms**	-	-	-	-	-	-	-	-	**0.01** *	1.89	1.19	2.98

**Bold:** * *p* < 0.05; CI, confidence interval.

## Data Availability

Data are available from the authors.
